# How Objects Are Grasped: The Interplay between Affordances and End-Goals

**DOI:** 10.1371/journal.pone.0025203

**Published:** 2011-09-28

**Authors:** Luisa Sartori, Elisa Straulino, Umberto Castiello

**Affiliations:** Dipartimento di Psicologia Generale, Università di Padova, Padova, Italy; University of Bologna, Italy

## Abstract

**Background:**

Substantial literature has demonstrated that how the hand approaches an object depends on the manipulative action that will follow object contact. Little is known about how the placement of individual fingers on objects is affected by the end-goal of the action.

**Methodology/Principal Findings:**

Hand movement kinematics were measured during reaching for and grasping movements towards two objects (stimuli): a bottle with an ordinary cylindrical shape and a bottle with a concave constriction. The effects of the stimuli's weight (half full or completely full of water) and the end-goals (pouring, moving) of the action were also assessed. Analysis of key kinematic landmarks measured during reaching movements indicate that object affordance facilitates the end-goal of the action regardless of accuracy constraints. Furthermore, the placement of individual digits at contact is modulated by the shape of the object and the end-goal of the action.

**Conclusions/Significance:**

These findings offer a substantial contribution to the current debate about the role played by affordances and end-goals in determining the structure of reach-to-grasp movements.

## Introduction

A number of studies on hand kinematics have demonstrated that how the hand approaches an object depends on the manipulative action that will follow object contact and grasping [1]–[7]. Marteniuk et al. [1] reported, for example, that they asked subjects to reach for an object and to either fit it into a similarly sized opening or to throw it away. Although the initial task requirement of reaching for an object was identical in the two conditions, kinematic analyses revealed substantial differences. Reaching movements performed in view of the “fit condition” had lower peak velocities and longer deceleration periods indicative of a more accurate approach phase compared with reaching movements in view of a “throw condition.” The influence of different consecutive motions during initial reaching and prehension movements was likewise recently examined by Armbrüster and Spijkers [5] who considered four after-grasp movements – lifting, raising, throwing and moving – which differed as far as direction and accuracy requirements were concerned. That study showed that movement parameter values are affected by the type of consecutive movement needed. Specifically, the peak aperture was larger and the peak deceleration was higher when the grasping was followed by a throwing or a placing movement with respect to a lifting motion. Ansuini et al. [6], [7] added a level of complexity to the analysis by considering if and how every fingers' angular excursion varied depending on the accuracy requirements of the action following grasping. By asking participants to grasp an object and to lift it to fit it into a tight opening or to place it in a large niche, the Authors showed that the degree that the end-goal of the action required accurate movements also affects the shaping of individual digits during the approach phase. On the basis of these findings, it would appear that what the individual intends to do with an object affects how the hand is shaped during the reaching movement.

Going one step further, it would seem that even hand placement on the object to be grasped is affected by the nature of the upcoming task following object contact. Cohen and Rosenbaum [4] asked participants to grasp a cylindrical object and to move it to a new position. They found that the point (height) on the cylindrical object where contact was made was inversely related to the height of the position to which it was to be moved. This result has been considered evidence that subjects anticipate the positions they will comfortably adopt once they have completed object transport movements. In other words, where people grasp objects can give insight about the movement that is being planned afterward.

Altogether, these results suggest that human hand movements are characterized by forms of movement that are linked to the end-goal of the action. To our knowledge, no studies have considered if individuals exploit the ability to choose where digits are placed to optimize end-goal requirements. To date, previous studies focusing on finger placement have all been characterized by single-step actions not entailing subsequent steps to complete the end-goal.

What remains largely unknown is, therefore, how the physical geometry of an object perceived and used for specific goals determines fingertip placement, a question connected to the notion of affordances first formulated by Gibson [8]. In his view, affordances should be considered properties reflecting the potential relationship between a subject and relevant aspects of the environment. As Gibson [8] explained, “…to see things is to see how to get about them and what to do or not to do with them.” In other words, the relevant aspects of the world are those used to guide actions.

**Figure 1:**
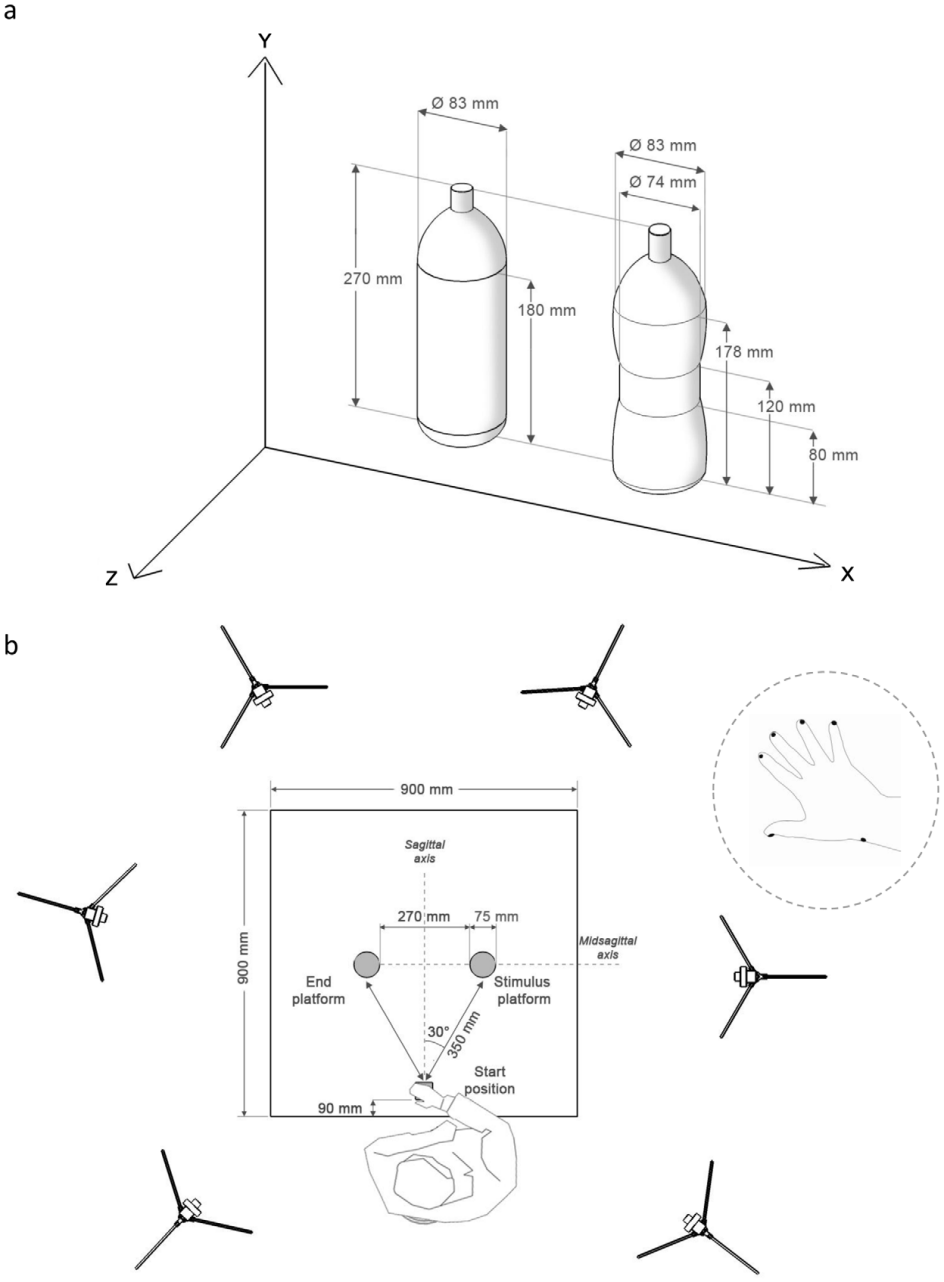
Graphical representation of the experimental stimuli and set-up. a. The two objects (stimuli) used in the experiments. To the left the ordinary shaped bottle. To the right the concave shaped bottle. b. Details of the experimental set up including the start position of the hand and relative distance from the stimulus platform and end platform positions are reported. In the inner circle the placement of infrared reflective markers on the wrist and the digits of participants is represented.

Although affordances have been studied in the context of reaching and grasping movements [9]–[18], how they interact with the end-goal of the action to determine a functional structure for reach-to-grasp movements before contact is made and the position that fingertips are placed on an object remain to be clarified. In our study we attempted to investigate how the shape and the weight of an object interact with end-goal requirements determining how the stimulus is approached and contacted. The object's shape was manipulated by utilizing one of two stimuli: a bottle with a regular cylindrical shape and another with a concave constriction (Fig. 1a). The object's weight was manipulated by presenting a completely full or a half full bottle of water. The end-goal was manipulated by requesting participants to move the stimulus to a new location or to pour some of its contents into a glass.

We wondered if a concave shape would result in a particular distribution of contact points regardless of the weight of the stimulus. Our question seemed reasonable in view of the fact that although symmetry is the rule in nature, in everyday life tools characterized by different mass centers, irregular shapes, and specific affordances are commonplace. How then are these ‘irregular’ features operationalized by the motor system to guide functional behavior?

## Materials and Methods

### Ethics statement

The experimental procedures were approved by the Institutional Review Board at the University of Padua and were in accordance with the Declaration of Helsinki (Sixth revision, 2008). All participants gave their informed written consent to participate in the study.

### Subjects

Thirteen volunteers (seven females and six males, between the ages of 21 and 34) with normal or corrected-to-normal vision participated in the experiment. All were right-handed according to the Edinburgh Inventory questionnaire [19] and were naive as to the experimental purpose of the study.

### Stimuli

Two differently shaped custom designed plastic bottles were utilized (Fig. 1a): an ordinary cylindrical shaped one (83 mm diameter; 270 mm height; Fig. 1a) and a second bottle of the same height and diameter with a concave constriction (74 mm in diameter extending 80 mm and 120 mm from the bottom of the bottle) (Fig. 1a). The height of the bottles was chosen to permit a wide range of vertical positions of the fingertips. Each bottle weighed 500 g when half full of water, and 1000 g when completely full of water.

### Experimental Setup

Figure 1b represents the experimental set-up. Each participant sat on a height-adjustable chair in front of a table (900×900 mm) with the elbow and wrist resting on the table surface and the right hand in the designated start position. In the start position, the hand was pronated with the palm resting on a pad (60×70 mm), which was shaped to allow for a comfortable and repeatable posture of all digits, i.e., slightly flexed at the metacarpal and proximal interphalangeal joints. The starting pad was attached 90 mm away from the edge of the table surface 50 mm away from the midsection. The bottle was placed on a round platform (75 mm diameter) which was located at a distance of 350 mm and an angle of 30° between the platform and the sagittal plane of the hand's starting position on the right side of the table. An end-platform (120 mm diameter) was located to the left of the participant at a distance of 350 mm and an angle of 30° between the pad and the sagittal plane of the hand's starting position. The pad was considered a landing platform during the trials in which the bottle was to be moved and to be placed on it, and it was considered a base for the glass in the trials in which the end-goal of the action was pouring.

### Kinematic recording

A 3D-optoelectronic SMART-D system (Bioengineering Technology & Systems, B|T|S|) was used to track the kinematics of the participant's right hand. Six light-weight infrared reflective markers (0.25 mm in diameter; B|T|S|) were placed on each participant's arm and hand (Fig. 1b, inner circle). One marker was placed on the wrist (the dorsodistal aspect of the radial styloid process) to measure the reaching component of the action. Five markers (referred to as “fingertips” in this manuscript) were placed on the central region of the nail of each of the digits to measure the grasping component of the action and the digits' placement on the object. A marker was also placed on the top of the bottle to track its movement after contact and to compute the location of each fingertip in relation to it. Six video cameras (sampling rate 140 Hz) detecting the markers were placed in a semicircle at a distance of 1–1.2 meters from the table. The camera position, roll angle, zoom, focus, threshold, and brightness were calibrated and adjusted to optimize marker tracking before the trials were begun. Static and dynamic calibration was then carried out. For the static calibration, a three-axes frame of five markers at known distances from each other was placed in the middle of the table. For the dynamic calibration, a three-marker wand was moved throughout the workspace of interest for 60 s. The spatial resolution of the recording system was 0.3 mm over the field of view. The standard deviation of the reconstruction error was 0.2 mm for the x, y, and z axes.

### Procedure

The participants were instructed to reach and grasp for the bottle and then to move it to a specific target position or to pour part of its contents into a glass. The shape (plain, concave) and the weight (half full or completely full of water) of the bottle as well as the end-goal (pouring, moving) of the action were varied. The participants were requested to start the action at the sound of a tone (880 Hz/200 ms) and each was tested in eight experimental conditions.

1. Half full ordinary shaped bottle, pouring;
2. Half full ordinary shaped bottle, moving;
3. Completely full ordinary shaped bottle, pouring;
4. Completely full ordinary shaped bottle, moving;
5. Half full bottle with a concave constriction, pouring;
6. Half full bottle with a concave constriction, moving;
7. Completely full bottle with a concave constriction, pouring;
8. Completely full bottle with a concave constriction, moving.

At the end of each trial, the participant was instructed to return the bottle to its original position and he/she returned to the start position. With reference to the ‘pouring’ condition, the bottle was refilled after the participant returned it to its original location. The participants were instructed to perform the tasks as naturally as possible. No other instructions were given with respect to how or where the stimulus was to be grasped. Each participant performed 10 trials for each experimental condition, for a total of 80 trials. Experimental conditions were randomized across participants. On the average, there was a time interval of about 15 seconds between trials. The experiment lasted approximately 40 min.

### Data processing

Following data collection, each trial was individually checked for correct marker identification and the SMART-D Tracker software package (B|T|S|) was used to provide a 3-D reconstruction of the marker positions as a function of time. The data were then filtered using a finite impulse response linear filter (transition band = 1 Hz, sharpening variable = 2, cut-off frequency = 10 Hz) [20], [21]. Movement onset was calculated as the time at which the tangential velocity of the wrist marker crossed a threshold (5 mm/s) and remained above it for longer than 500 ms. Stimulus lift onset was calculated as the time at which the tangential velocity of the top center marker of the stimulus crossed a threshold (5 mm/s) and remained above it for longer than 200 ms. Digit contact time was defined as the time at which the digits made contact with the stimulus from the moment the movement was begun and quantified as the time at which the tangential velocity of the fingertip marker reached its minimum value. The algorithm's accuracy in determining digit contact time was verified by previous studies [e.g., 22]. At that point we wrote a custom algorithm to compute the following temporal and spatial variables proven to be specifically relevant to the hypotheses being tested [6], [22]. The time interval between movement onset and digit contact time (movement time), the time at which the tangential velocity of the wrist was maximum from movement onset (time of maximum wrist velocity), the time at which the distance between the three dimensional coordinates of the thumb and index finger was maximum between movement onset and digit contact time (time of maximum grip aperture), the maximum distance reached by the three dimensional coordinates of the thumb and index finger (grip aperture), and the x- and y-coordinates (horizontal and vertical dimensions, respectively) of the fingertips upon the object at contact time (fingertip contact points). The contact point x-coordinate refers to the horizontal distance between the fingertip and the origin of the axes. The y-coordinate was defined as the vertical distance (in millimeters) between the fingertip and the base of the cylinder. Note that the third dimension (z) of the fingertip coordinate in contact with the object is redundant because it covaries with the x-coordinate. The measurements were made along the three Cartesian axes [i.e. X (left-right), Y (up-down), and Z (anterior-posterior) axes] of the participants in an upright sitting position. Data processing was confined to the reach-to-grasp phase (from the movement onset until the moment the fingers made contact with the bottle), the phase common to all the experimental conditions. We investigated whether hand movements were modulated during that phase by the weight or shape of the object to be grasped or by the end-goal of the action.

### Data analysis

The mean value for each parameter of interest of the eight experimental conditions were determined for each participant and then entered into a repeated-measures ANOVA with the ‘stimulus shape’ (ordinary, concave), ‘weight’ (completely full or half full) and ‘goal’ (pouring, moving) as the within-subject factors. Preliminary analyses were conducted to check for normality, sphericity (Mauchly test), univariate and multivariate outliers, with no serious violations noted. Main effects were used to explore the means of interest (*post hoc t*-test), and Bonferroni's corrections (alpha level of *p*<0.05) were applied.

## Results

### Movement time

**Figure 2:**
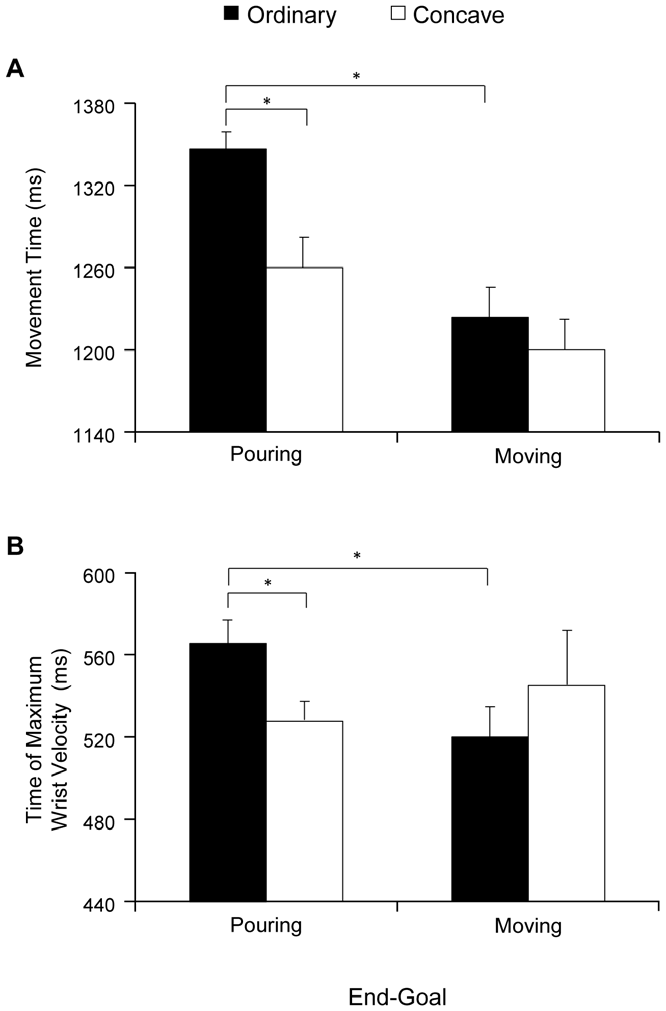
Graphical representation of the interaction shape x goal for key kinematic parameters for the reaching component. a. Movement time; b. Time of maximum wrist velocity.

The ANOVA performed on movement time revealed a significant main effect of stimulus shape [F_(1,12)_ = 10.75, *P*<0.05]. Movement time was longer for the ordinary than for the concave object [1288 vs. 1244 ms, respectively). The main effect of weight was likewise significant [F_(1,12)_ = 24.79, *P*<0.001]. Movement time was significantly shorter when the object was half full compared to completely full (1221 vs. 1311 ms, respectively). The main effect of the end-goal was also significant [F_(1,12)_ = 54.14, *P*<0.001]. Movement time was longer when the end-goal of the action was pouring rather than moving (1308 vs. 1224 ms, respectively). There was also a significant interaction between shape and end-goal [F_(1,12)_ = 5.95, *P*<0.05; Fig. 2a]. Post-hoc comparisons revealed that when the end-goal was pouring, movement time was significantly shorter for the concave than for the ordinary shaped object (*P*<0.05). Furthermore, while the movement time was longer for the ordinary shaped object when the end-goal was pouring rather than moving (*P*<0.001), the difference was not statistically significant for the concave object. The other differences were not statistically significant.

### Time of maximum wrist velocity

The ANOVA performed on the time of maximum wrist velocity revealed a significant interaction between shape and end-goal [F_(1,12)_ = 10.00, *P*<0.05; Fig. 2b]. Post-hoc comparisons showed that when the end-goal was pouring and the object was concave, the time of maximum wrist velocity was significantly anticipated with respect to that for the ordinary shaped object (*P*<0.05). Furthermore, while the time of maximum wrist velocity was longer for the ordinary shaped object when the end-goal was pouring rather than moving (*P*<0.05), the difference was not statistically significant for the concave object. The other differences were not statistically significant.

### Time of maximum grip aperture

**Figure 3:**
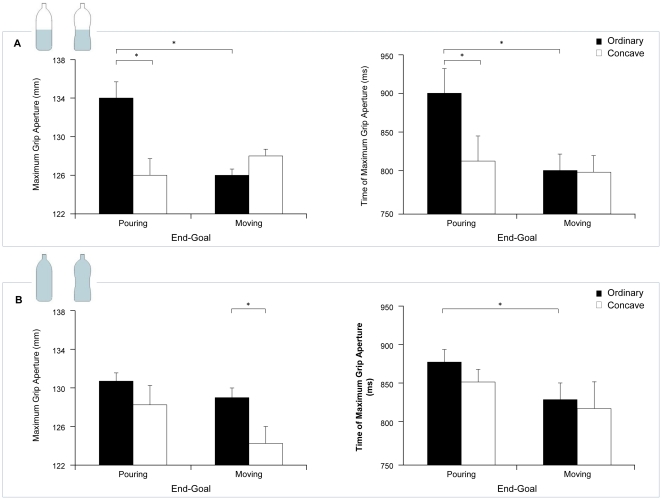
Graphical representation of the interaction shape x goal x weight for key kinematic parameters for the grasping component. a. Time and amplitude of the maximum grip aperture for the half full bottle; b. Time and amplitude of maximum grip aperture for the completely full bottle.

The ANOVA performed on the time of maximum aperture revealed a significant main effect of shape [F_(1,12)_ = 8.15, *P*<0.05]. The time the peak grip aperture occurred was later for the ordinary shaped than for the concave bottle (851 vs. 821 ms, respectively). The main effect of the end-goal was likewise significant [F_(1,12)_ = 17.46, *P*<0.001]. The time the peak grip aperture occurred was later when the end-goal of the action was pouring rather than moving (860 vs. 812 ms, respectively). The interaction between shape, end-goal, and weight was also significant [F_(1,12)_ = 5.68, *P*<0.05; Fig. 3a,b]. Post hoc comparisons revealed that the time the index and thumb reached the maximum distance was significantly anticipated for the concave bottle with respect to the ordinary shaped one (*P*<0.05) only when the end-goal was pouring and the bottle was half full (Fig. 3a). Furthermore, while the maximum grip aperture was later when the end-goal was pouring rather than moving for both the half full and the completely full ordinary shaped object (*P_s_*<0.001), the difference was not statistically significant for the concave bottle. The other differences were not significant.

### Maximum grip aperture

The ANOVA performed on grip aperture revealed a significant main effect of shape [F_(1,12)_ = 5.77, *P*<0.05]. The distance between the index and the thumb reached a smaller peak when the bottle was concave with respect to the ordinary shaped one (126 vs. 130 mm, respectively). Moreover, a significant interaction between shape, end-goal, and weight [F_(1,12)_ = 8.10, *P*<0.05] showed that when the end-goal was pouring and the stimulus was half full, the grip aperture was significantly smaller for the concave than for the ordinary shaped bottle (*P*<0.05; Fig. 3a). When the end-goal was moving and the object was completely full, the grip aperture was significantly smaller for the concave with respect to the ordinary shaped bottle (*P*<0.05; Fig. 3b). Furthermore, while the maximum grip aperture was greater when the end-goal was pouring rather than moving for the half full ordinary shaped object (*P*<0.001; Fig. 3a), the difference was not statistically significant for the concave bottle. The other differences were not significant.

### Fingertip contact points

The separate ANOVAs performed on the *x* and *y* coordinates for each digit indicate that the object's weight had a similar effect on all the digits, but the position of the contact points of all the fingers was specifically modulated as a function of the object's shape, the end-goal of the action, and their relationship.

#### Effect of weight

A significant main effect of weight for all the fingers was found (for mean and statistical values refer to Table 1). For the *x* axis, all the fingers except the thumb were placed more internally towards the center of the mass of the completely full than of the half full object. For the *y* axis, the significant main effect of weight indicated that all the fingers were placed lower for the half full rather than the completely full bottle.

**Table 1:** Mean and statistical values for the main effect of weight for all the fingers.

		*Half full*	*Completely full*	*Statistical Values*
Thumb	*x*	220 mm	213 mm	F_(1,12)_ = 2.79, p>0.05
	*y*	120 mm	130 mm	F_(1,12)_ = 34.96, p<0.001**
Index	*x*	215 mm	210 mm	F_(1,12)_ = 13.03, p<0.05*
	*y*	147 mm	158 mm	F_(1,12)_ = 19.78, p<0.001**
Middle	*x*	202 mm	191 mm	F_(1,12)_ = 24.78, p<0.001**
	*y*	111 mm	123 mm	F_(1,12)_ = 37.28, p<0.001**
Ring	*x*	208 mm	196 mm	F_(1,12)_ = 24.82, p<0.001**
	*y*	86 mm	97 mm	F_(1,12)_ = 34.18, p<0.001**
Little	*x*	221 mm	213 mm	F_(1,12)_ = 8.84, p<0.05*
	*y*	51 mm	64 mm	F_(1,12)_ = 29.41, p<0.001**

#### Effect of shape

**Figure 4:**
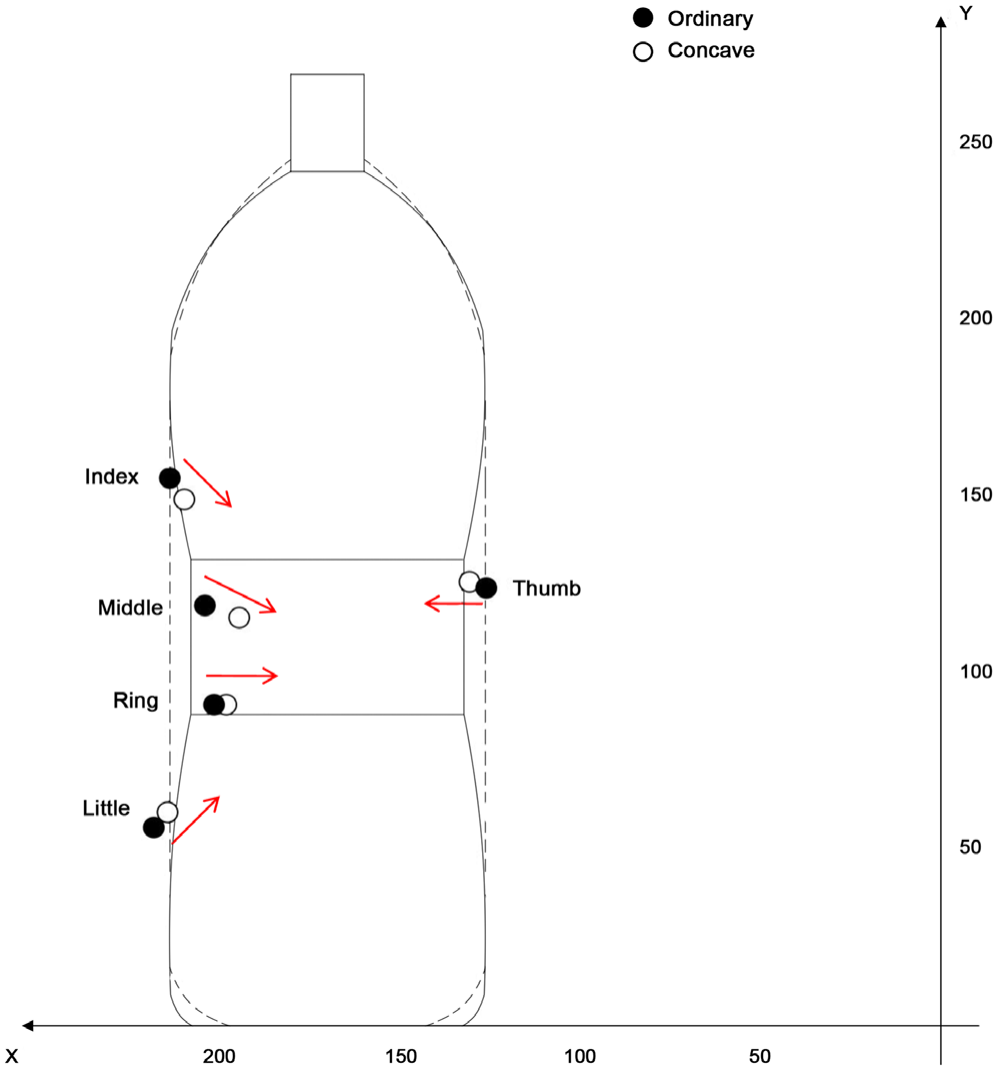
Graphical representation of the main effect stimulus shape for contact points. The distribution of fingertip placement on the stimuli is described. Arrows indicate how the fingertips are moved from the ordinary (black dots) to the concave (white dots) stimulus. Note how the fingertips at the extremity of the concave stimulus (i.e., index and little fingers) move closer to the constriction and contribute to the digits' orientation towards the center of the mass.

For the *x* axis a significant main effect of object shape was found for all the fingers: the thumb [F_(1,12)_ = 18.29, *P*<0.001], the index [F_(1,12)_ = 10.69, *P*<0.05], the middle [F_(1,12)_ = 32.87, *P*<0.001], the ring [F_(1,12)_ = 4.75, *P*<0.05], and the little [F_(1,12)_ = 5.28, *P*<0.05] fingers were all grouped at the point the concave stimulus was constricted. That action along the *x* axis brought the fingers and the thumb closer to the object's center of mass (Fig. 4). Specifically, the index finger was placed more internally with respect to the center of mass for the concave rather than for the ordinary shaped bottle (210 vs. 214 mm, respectively). The same was true for the middle (195 vs. 204 mm, respectively), the ring (197 vs. 202 mm, respectively), the little (215 vs. 218 mm, respectively) fingers, and the thumb (131 vs. 127 mm, respectively). For the *y* axis, a significant main effect of object shape was found for the index [F_(1,12)_ = 13.39, *P*<0.05] and the little [F_(1,12)_ = 5.28, *P*<0.05] fingers. The index finger was placed lower towards the constriction for the concave with respect to its placement on the ordinary shaped object (149 vs. 155 mm, respectively). Analogously, the little finger was placed higher – within the constriction – for the concave bottle than for the ordinary shaped one. But no main effect of shape was observed for the thumb, the ring, and the middle finger and this was because, as shown in Figure 4, they were already comfortably placed within the concavity in terms of the *y* coordinates.

#### Effect of the end-goal

**Figure 5:**
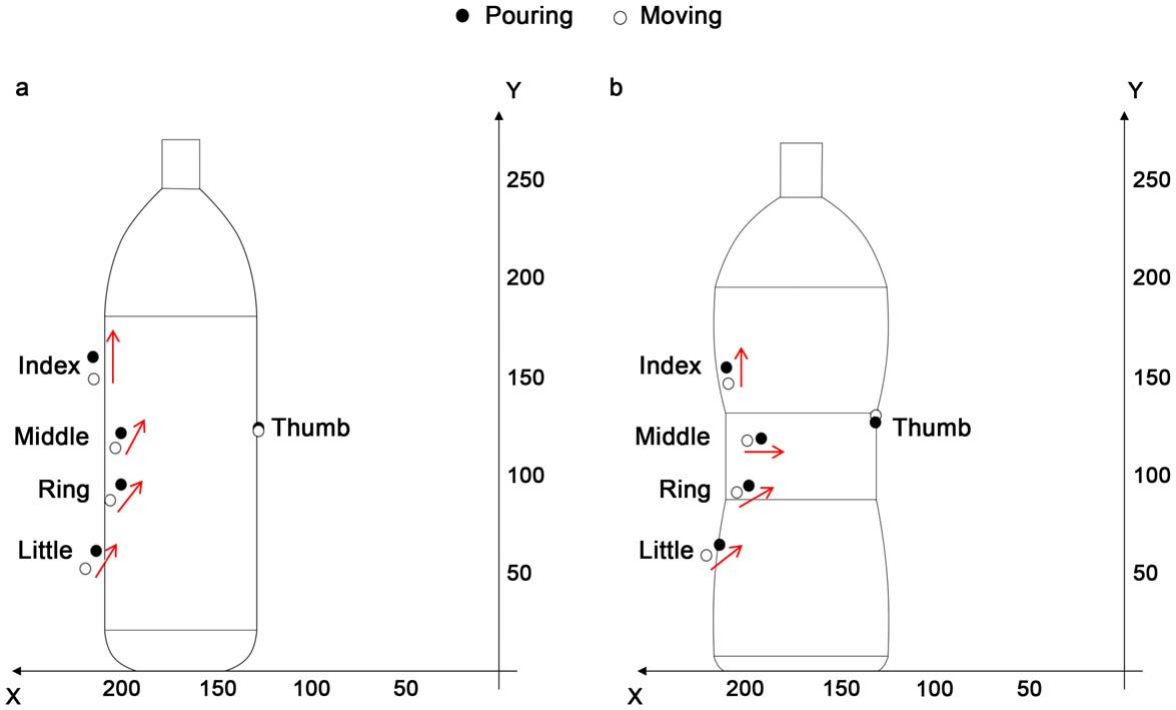
Graphical representation of the main effect of the end-goal for contact points. a. The distribution of fingertips upon the ordinary shaped stimulus; b. The distribution of fingertips on the concave stimulus. Arrows represent how the fingertips are shifted from the ‘pouring’ (black dots) to the ‘moving’ (white dots) goals. Note how the pouring action triggers the approaching of the middle finger and ring towards the thumb, while the remaining fingertips (i.e., index and little fingers) are shifted higher for greater control.

**Figure 6:**
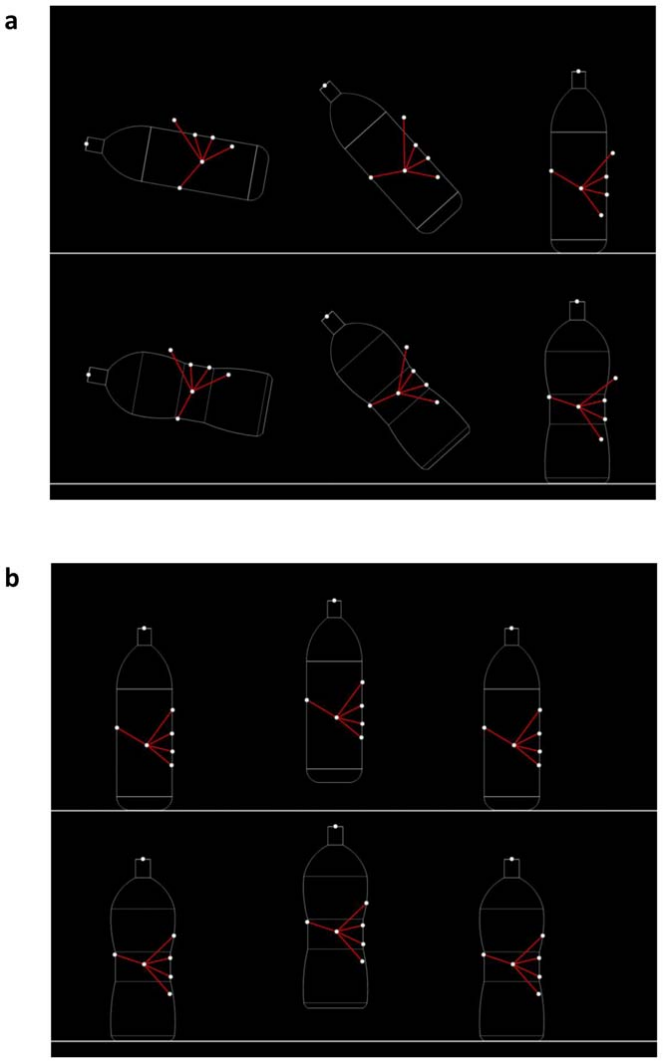
Graphical representation for the ‘index finger’ effect. A. Distribution of fingertip placement during the ‘pouring’ action end-goal for both the ordinary (a) and concave (b) stimuli. B. Distribution of fingertip placement during the ‘moving’ end-goal for both the ordinary (a) and concave (b) stimuli. Note that for the pouring action the index finger is placed external to the stimulus surface.

For the *x* axis a significant main effect of the end-goal was found for the middle [F_(1,12)_ = 7.58, *P*<0.05] and the ring [F_(1,12)_ = 6.24, *P*<0.05] fingers. Both were placed more internally with respect to the object's center of mass when the end-goal was pouring rather than moving (Fig. 5a,b). For the *y* axis, the main effect of the end-goal was found for the index [F_(1,12)_ = 10.85, *P*<0.05], the ring [F_(1,12)_ = 5.28, *P*<0.05], and the little [F_(1,12)_ = 10.68, *P*<0.05] fingers, all placed higher when the end-goal was pouring rather than moving (Fig. 5a,b). On the contrary, neither the thumb nor the middle finger were modulated by the end-goal and their position remained constant for both end-goals. A significant interaction between the shape and the end-goal was found for the index finger [F_(1,12)_ = 5.94, *P*<0.05], indicating that when the end-goal was pouring, it was placed lower on the concave than on the ordinary shaped object (*P*<0.05). Interestingly, it was observed that all the participants adopted a particular ‘index finger pattern’ depending on the end-goal. For the pouring condition, the index finger tended to move away from the surface of the stimulus. As a result we conducted a series of ANOVAs similar to those described above considering the 3-D distance between digits. The main effect of the end-goal was significant for the distance between the thumb and the index finger [F_(1,12)_ = 36.02, *P*<0.001]. The distance was increased when the end-goal was pouring rather than moving (106 vs. 99 mm, respectively; Fig. 6a,b). Similarly, the main effect of the end-goal was significant for the distance between the middle and the index fingers [F_(1,12)_ = 23.61, *P*<0.001] which was greater when the end-goal was pouring rather than moving (48 vs. 37 mm, respectively).

## Discussion

This study investigated how object affordances affect the kinematic structure of reach-to-grasp movements, with particular reference to finger contact points. Our findings suggest that it was the functional interaction between object affordances and end-goals that determined significant kinematic variations as far as how an object was approached and how and where fingers were placed on it. Only the concave shaped object produced a *facilitatory* effect for the reaching component in terms of both a shorter movement duration and an anticipated wrist peak velocity when the goal was pouring rather than moving. The time and amplitude of the maximum grip aperture were earlier and lower for the grasping component of a concave rather than ordinary shaped stimulus when the goal was pouring rather than moving. This effect was more evident when the stimulus was half full instead of completely full. The distribution of contact points have been found to vary in relation to different shapes and end-goals.

### Kinematics before stimulus contact

#### Effect of end-goal

Reach-to-grasp actions have been studied extensively to investigate the functional relationship between the properties of graspable objects and movements' kinematic aspects [e.g.], [ 23]–[25]. Recent studies have also demonstrated that the kinematics of reach-to-grasp actions prior to contact vary depending on the purpose of the action [1], [5]–[7]. According to previous data, what will take place after a grasping movement has a specific effect on the temporal aspects of reach-to-grasp movements. It was found, for instance, that movement duration is longer when the end-goal is a pouring action, similar to the pouring action considered in the present study [6], [7]. This result can be explained in terms of the degree of precision necessary to carry out the end-goal of the action. Consistent with these findings, we found that the action of pouring determined a longer movement duration.

#### Effect of weight

Differences in the type of stimuli and end-goals were evident only when the stimulus was lighter, and in that case the time and the amplitude of the maximum grip aperture occurred earlier and were lower for the concave than for the ordinary shaped object when the goal was pouring rather than moving. This would suggest that object dynamics are influenced by end-goal requirements. By combining knowledge regarding stimulus dynamics, object geometry, and end-goals, the motor system might be able to predict the load force and to adjust grip force accordingly [26], [27]. When the concave object was lighter the constraints on grip load were lower possibly permitting the affordance provided by the object's shape to be considered. Anticipating the maximum grip aperture may have provided more time during the grasp closing phase giving the subject the opportunity to choose with greater accuracy where to place the fingers on an irregularly shaped object. A lower amplitude of maximum grip aperture may indicate that the subject was, from the very beginning of the action, already aiming towards the concavity. Conversely, when the stimulus was heavier, and the object was more likely to tilt over, load force prediction and computation may have taken precedence over kinematic patterning. Put simply, planning stimulus dynamics may have become more demanding. Adjusting to a non-spatial property such as weight is complicated since it is not enough to rely on low-level visual features alone, but the visual appearance of the object must also be related to memory representations [28]–[36]. When weight-related demands increase, then, the visual cue receiving the most attention is the one concerned with stimulus dynamics rather than stimulus geometry.

#### The interaction between shape affordance, end-goal and weight

The strong manipulative affordance provided by a concave shaped object drastically reduced the time for the ‘pouring’ movement, bringing it to the level of the moving action. The object's affordance produced, then, a somewhat *facilitatory effect* which diminished the constraints dictated by the greater precision needed for the end-goal. Interestingly, while it was only the shape of the stimulus that interacted in a significant way with the end-goal when the reaching component was concerned, the interaction also involved the objects' weight when the grasping component was examined.

### Distribution of contact points

Previous studies on affordance in relation to reach-to-grasp movements have addressed how an object's physical geometry [11], [17] together with other properties such as its center of mass [22], [37] determine grasp point selection. Our results take these observations a step further by indicating that participants exploited the object's shape to choose the grasp point on the basis of the end-goal of the action.

In fact, the index, the ring and the little fingers were functionally modulated in our participants depending on the end-goal of the action, while the thumb and the middle finger were not. The former were placed higher on the object when the goal was pouring rather than moving, possibly allowing for greater control needed to execute a pouring action. For the concave-shaped stimulus, they were grouped around the constriction closer to the center of the mass. It is possible that the object's shape determined an affordance-based ‘grouping’ which overrode weight-related visual cues and end-goal requirements. This is important because it reveals how contact points reflect the interplay between the actor and the object geometry, that is, affordances considering also that no instructions were given to the participants about where they were to grasp the bottles.

Moreover, although weight seemed to modulate the contact point position, as previously reported [e.g., 38], it did not interact with the other variables considered here. Rather, a main effect of weight emerged suggesting that for heavier stimuli all the fingers move to a higher position regardless of the object's shape and the end-goal of the action. Again, as mentioned above, this movement might be functional to permit greater control when stimulus dynamics become more demanding.

When modulation of the contact points was examined on a digit-to-digit basis, we observed that the index finger never contributed to execution of the task at hand. Indeed, all the participants placed the index finger external to the stimulus which seemingly served as a stabilizing mechanism to minimize object roll. Analysis of the 3-D distance between the index finger and the other digits firmly grasping the stimulus showed that it increased when the task was pouring rather than moving, suggesting that the greater the precision needed by the task, the more the index finger is placed externally. Such modulation for the index finger is in line with the increased vertical distance between the thumb and index finger for pouring but not for lifting reported by Crajè and colleagues for a stimulus similar to ours [39]. These observations seem to provide evidence of the distinctive roles played by the different fingers. To some extent, the index finger can be regarded the “navigator” assisting the thumb, which can be considered the “pilot” [40], [41], during computation of a hand trajectory towards a target. The thumb position was notably unaltered.

Careful placement of the index finger on the object has always been reported as a prerequisite for a stable grasp to minimize object roll and is based on evidence that the thumb and index finger have a stronger force production capability compared to the other digits [42], [43]. Iberall [16] accordingly introduced the concept of an “opposition axis” extending between the thumb and finger(s) defining a relevant unit of analysis to organize and control grasping. Since then the opposition axis has become a commonly accepted concept by investigators studying the effects of affordances on reach-to-grasp movements [32]. Our findings have shown that when the task is whole-hand grasping, such as for pouring, the action is centered on the opposition axis formed by the thumb and the middle finger. It is noteworthy that the majority of studies on this subject have been limited to precision types of grasping involving only the thumb and index finger and not linked to end-goals requiring a subsequent step. It is possible then that the ‘index finger’ effect takes place when no specific instructions on fingertip placement on objects are given and when the end-goal requires a stabilizing mechanism. In the present study, the constriction in one of the bottles might have affected the choice with regard to the opposition axis. While this factor was not the primary object of our study, our results suggest that it is the opposition axis between the thumb and middle finger that is an unexpected finding. The hypothesis can be made that the behavior of digits involved in the opposition axis may be modulated by specific contingencies.

In the light of the tasks executed during our trials, it would seem that relieving the index finger of its role in the opposition axis could promote its re-employment as a “navigator” during the computation of complex hand trajectories. Additional studies will be able to investigate the role of the opposition axis in relation to affordances and end-goal constraints.

### Conclusions

Our study reveals that effective manipulation of objects depends on the individual's ability to perceive object affordances. Specifically, our results suggest that when subjects can choose contact points and object affordances are available, they implement functional mechanisms to control grasping through a careful selection of fingertip placement. A strong visual affordance can override task constraints such as those dictated by the object's weight and end-goal requirements. Both ‘prior to contact’ and ‘contact’ findings support the hypothesis that affordances play a role in determining the structure and the scaling of reach-to-grasp actions [18]. They sustain the hypothesis of an “end-goal functional affordance-based” model. Manipulation of the affordances made available within a task causes specific changes in key kinematic landmarks of the reach-to-grasp structure depending on the end-goal of the action. When it is freed from dynamics constraints, ‘shape’ affordance appears to be pivotal in determining the reach-to-grasp structure. When the end-goal requires a greater level of accuracy and calls for a more precise computation of stimulus dynamics, ‘weight’ affordance become more relevant.

An interesting issue stemming from the present findings is whether affordances are represented or whether they are in-built in the visual flaw. The basis for such investigation should rely on the evidence that visual objects potentiate actions that might be performed on them [44]–[46]. And on the notion of dual, independent routes to action from seen objects [47]. One route mediated by visual activation of semantic-functional knowledge about objects. Another route mediated solely by visual information derived from the objects. Additional studies investigating the nature of object affordances and their relationship with end-goals are of course warranted.
